# From Shattered Goals to Meaning in Life: Life Crafting in Times of the COVID-19 Pandemic

**DOI:** 10.3389/fpsyg.2020.577708

**Published:** 2020-10-15

**Authors:** Elisabeth M. de Jong, Niklas Ziegler, Michaéla C. Schippers

**Affiliations:** Department of Technology and Operations Management, Rotterdam School of Management, Erasmus University, Rotterdam, Netherlands

**Keywords:** COVID-19, pandemic, life crafting, grief, collective trauma, meaning in life, purpose in life, goal setting

## Abstract

The novel COVID-19 pandemic has created an extraordinary situation for our generation, with many countries being on lockdown. With this, new situation comes many psychological challenges not only for health care workers and people suffering from COVID-19 but also for the general population. Adapting to the new situation can be demanding. Experts have suggested that emotions during this situation are very similar to grief, and people experience emptiness and sadness about the loss of their normal lives, which can even lead to a loss of meaning in life. In this paper, we argue that life crafting could offer a way to help people cope with the situation and renew their sense of meaning. A life crafting intervention is based on theoretical insights from multiple areas of research, like positive psychology, expressive writing, and the salutogenesis framework. Life-crafting interventions help people find meaning in life by focusing on their ideal future, and helping them set goals, and make concrete plans to achieve those goals and overcome obstacles. Since having a clear purpose or meaning in life has been shown to have many benefits, we propose that it can also help people to cope with the psychological effects of the pandemic. A life-crafting intervention can offer people a chance to evaluate their goals in a time of uncertainty and rediscover meaning in life to guide them through these difficult times.

## Introduction

The COVID-19 pandemic has caused a unique situation in the world. There are many different measures being taken to contain the virus. Most countries around the world have implemented a “lockdown” in some form, and although some countries have stricter regulations than others, most of them involve at least some type of so-called “social distancing” (Hale et al., 2020). In a short period of time, the normal life that people were used to living has been drastically and unexpectedly changed. This has consequences for people’s mental and physical well-being (for a review, see Schippers, 2020).

Grief experts have suggested that emotions during the COVID-19 pandemic are very similar to grief, as in the case of losing a loved one (Berinato, 2020). Kessler described the current situation as follows: “Our world as we knew it has died and we are feeling the sadness” (Amanpour and Company, 2020; Berinato, 2020). In accordance with these statements, scientific research has also shown that grief is not only experienced after a bereavement but can also play a role after other life changing losses, such as a divorce or job loss (Papa et al., 2014). Although these forms of grief are rather individual, more collective forms of grief that are not necessarily related to direct individual experiences of bereavement can also occur, for example, in refugees when they need to adjust to a host country (Baškauskas, 1981).

There are several ways grief might play a role during the COVID-19 pandemic. Needless to say, people who are directly affected by the virus or have loved ones who have suffered from or even passed away because of the virus experience grief. However, these grief processes are not the focus of this paper. Rather, this paper is directed at the collective grief processes that might be present in the general population, as a result of a loss of normalcy, caused partly by the many containment measures. This loss of normalcy and the grief over what is no longer possible can lead to a sense of emptiness, and even a loss of meaning in life (Berinato, 2020; Taha, 2020). Some researchers have even suggested that isolation measures that take more than 10 days may lead to post traumatic stress syndrome (Schippers, 2020). In accordance with this, different theories have shown that finding meaning is an important element for recovery in a grief process, and have suggested that it can help in finding post-traumatic growth instead of post-traumatic stress (Hogan and Schmidt, 2002; Janoff-Bulman, 2006; Updegraff et al., 2008; Kessler, 2019). As the mental health effects can be quite severe (Fegert et al., 2020; Schippers, 2020), restoring a sense of meaning in life can be an essential part of the healing process (e.g., Hogan and Schmidt, 2002; Updegraff et al., 2008). However, research also suggests that people might need guidance to find meaning in a structured manner (Steger et al., 2008). Therefore, in this perspective paper, we argue that a life crafting intervention, which is aimed at finding meaning in life, could be helpful to guide people through this grief-like process.

## Grief and Finding Meaning

Finding meaning seems to be a central theme in the grief and trauma literature. However, the term “meaning” has been defined and operationalized differently across different fields of study. In their review, Martela and Steger (2016, p. 531) distinguished between three main types of meaning in life: coherence, purpose, and significance. Coherence refers to “a sense of comprehensibility and one’s life making sense.” Purpose means having “a sense of core goals, aims, and direction in life,” and significance refers to “a sense of life’s inherent value and having a life worth living”.

In the literature about grief and trauma, finding meaning often refers to the first type of meaning, coherence, conceptualized as making sense of what has happened. One well-known theory in the literature on grief and trauma is the theory of shattered assumptions, developed by Janoff-Bulman (1992). According to this theory, there are three fundamental human assumptions about the self and the world that form a person’s assumptive world, and that guide our day-to-day thoughts and behaviors. These assumptions are that the world is benevolent and meaningful, and that the self is worthy. A traumatic event can shatter these fundamental assumptions. To recover, assumptions should be rebuilt. One way to do this is to find meaning in the traumatic event, or, in other words, a way to make sense of it. Schwartzberg and Janoff-Bulman (1991) showed that the greater the ability of a bereaved individual to find meaning, defined as making sense of the loss, the less intense their grief. Although this theory is usually referred to in studies about individual grief or trauma, research by Updegraff et al. (2008) showed that finding meaning is also of importance after a collective trauma, in this case the 9/11 terrorist attacks. They found that in the general population (i.e., the majority of their sample consisted of people who were not directly exposed to the attacks), finding meaning, again defined as making sense of what happened, in the early aftermath of the event was related to lower post-traumatic stress symptoms in the 2 years following. This effect was mediated by reduced fears of future terrorism, which the authors saw as a sign that finding meaning led to rebuilding of assumptions about security and invulnerability. This definition of meaning thus refers to finding meaning in the events that have occurred and rebuilding assumptions of a meaningful and coherent world.

Another kind of meaning that seems important in the grief process is the meaning in one’s own life, which corresponds more with meaning in the sense of purpose and significance, as defined by Martela and Steger (2016). Besides making sense of the event itself and rebuilding assumptions about the world, rebuilding the assumptive world seems to entail more. Janoff-Bulman (2006) also suggested that rumination about questions regarding the meaning of life itself may later shift to rumination about finding meaning in one’s own life. In general, having a clear sense of purpose in life has been shown to have many benefits for mental as well as physical well-being (for a review, see Schippers and Ziegler, 2019). In the context of trauma, Sawyer and Brewster (2019) also showed that meaning in life was positively related to post-traumatic growth after bereavement. In the specific context of the COVID-19 pandemic, Trzebiński et al. (2020) have shown that a higher level of meaning in life (i.e., having a clear purpose and meaning in life, having life goals, not being afraid of the future; comparable to what Martela and Steger define as “purpose”) was related to lower anxiety and emotional distress during the crisis. Therefore, the authors argue that meaning in life (i.e., purpose), among other factors, may work as a buffer against stress reactions to the pandemic. Notably, whereas they assessed meaning in life as a stable factor, the authors argue that in the face of a prolonged crisis, meaning in life may be affected as well.

In the present paper, we predominantly focus on the second and third type of meaning as distinguished by Martela and Steger (2016): purpose and significance. In line with the reasoning of Trzebiński et al. (2020), we expect that the sense of purpose and in severe cases even significance in life for many people in the general population might have already been affected during the pandemic. The UN agency has estimated that in the second quarter of 2020, 305 million jobs have already been lost worldwide, mainly caused by prolonged containment measures (Straus, 2020). Furthermore, the IMF has predicted a severe worldwide economic crisis (International Monetary Fund, 2020). In addition, as described earlier, grief-like emotions over the loss of normalcy can also lead to a loss of purpose in life. In accordance with this, one study has shown that during the COVID-19 pandemic, the sense of purpose in life of students in higher education decreased in the second half of the academic year, whereas in the cohort of students from the year before, it remained stable (Schippers et al., in preparation).

We expect that there are individual differences in the degree to which purpose and significance are affected by the pandemic and the containment measures. For some people, life may have remained relatively normal, and their purpose in life may have stayed intact. However, because of the containment measures, some of their underlying goals might have been compromised. For example, someone’s purpose in life might be to become a psychologist, but because of the containment measures they cannot do their internship as planned (i.e., cannot attain this intermediary goal), and they need to find new ways and set new goals to reach their purpose. For others, who have, for example, lost their job or even their company (i.e., their life’s work) during the pandemic, their purpose or even significance in life itself might also be harmed. Consequently, the individual might experience a loss of directionality in their life as “goal are signals that orient a person to what is valuable, meaningful and purposeful” (Emmons, 2003, p. 107) and can be seen as a key element in human functioning (Emmons, 2003; Schippers and Ziegler, 2019). Some might even lose their sense of significance in life. Purpose and significance in life are often entangled. Significance is partly dependent on purpose, but also on other factors such as relationships with friends or family (Martela and Steger, 2016). However, since the containment measures mainly comprise of social distancing, this may make it more difficult to maintain social connections and support, which potentially makes the threat to the sense of significance even larger. Since many studies have shown that having purpose in life is essential to well-being and health (e.g., Hill and Turiano, 2014; Kim et al., 2014; for a review, see Schippers and Ziegler, 2019), we argue that it should be rebuilt. We propose that a life-crafting intervention could help people in rebuilding their sense of purpose and significance in life.

## What Is Life Crafting and How Can it Help to Find Meaning?

Individuals searching for meaning are often unlikely to do so in an organized manner and might be more focused on the past and present than particularly concerned about the future (Steger et al., 2008). Relatedly, while the presence of meaning in life is associated with positive outcomes, the actual (prolonged) search for meaning is associated with greater negative outcomes, and such a search could be indicative of meaninglessness (Updegraff et al., 2008; Linley and Joseph, 2011).

A more structured approach to finding meaning and purpose in life, called “life crafting,” was recently proposed by Schippers and Ziegler (2019, p. 3). They defined the term life crafting as “a process in which people actively reflect on their present and future life, set goals for important areas of life – social, career, and leisure time – and, if required, make concrete plans and undertake actions to change these areas in a way that is more congruent with their values and wishes.” Subsequently, the authors discuss an expressive-writing intervention to aid individuals in finding a purpose in life, while at the same time ensuring that they make concrete plans to work toward this purpose. This type of expressive writing exercises has shown to have benefits for (mental) health as well as academic performance (e.g., Lepore and Smyth, 2002; Morisano et al., 2010; Morisano and Shore, 2010; Schippers et al., 2015, 2020), and has roots in the fields of positive psychology, expressive writing (King and Pennebaker, 1996; Pennebaker, 1997; King, 2001), and salutogenesis (Antonovsky, 1996). Participants usually take part in such a life crafting intervention *via* an online questionnaire that guides them through the different writing exercises (e.g., Schippers et al., 2015), but could also be delivered by a chatbot (Dekker et al., 2020). A central part of the life crafting intervention described by Schippers and Ziegler (2019) is based on the Japanese concept of “Ikigai;” which can be defined as a sense of “a life worth living” (Sone et al., 2008, pp. 709). The term ikigai directly relates to the significance of one’s life, which has been defined as the third facet of meaning in live, next to purpose and coherence (Martela and Steger, 2016, pp. 537). As the authors describe, significance “is about evaluating one’s life as a whole, including past, present, and the future, while the other (purpose) is distinctively future-oriented: it is about evaluating the potential future value of one’s life through sustained goals that give life direction and momentum”. As such, the life-crafting intervention proposed by Schippers and Ziegler (2019) does not only strive to provide a framework which can help the individual in structuring their search for a (renewed) purpose in life but also lets the individual reintegrate this new purpose into their life as a whole (significance).

### How Can Life Crafting Help to Find Meaning During the COVID-19 Crisis?

**Figure 1:**
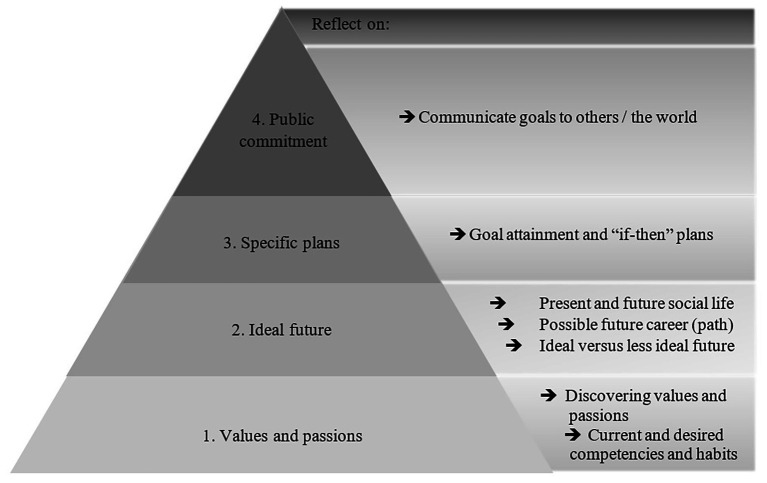
Overview of a life-crafting intervention (adapted from Schippers and Ziegler, 2019).

Important elements of a life-crafting intervention are: (1) discovering values and passions, (2) reflecting on one’s ideal future, (3) writing about specific goal attainment and “if-then” plans, and (4) making public commitments to the goals set (Schippers and Ziegler, 2019; see also Table 1 and Figure 1). In general, people often have difficulty with finding meaning in life, and therefore, a life-crafting intervention could be beneficial to many people. As it seems that the timing of interventions is crucial (Wilson, 2011), this may be particularly useful when people experience a loss of meaning. For the current pandemic situation, we propose several adjustments to the original intervention. First, it should be assessed what exactly has been shattered for the individual. Is it just their goals, or also their purpose in life or even their sense of significance in life? Second, based on this assessment, a custom intervention could be presented to the individual. For individuals with compromised goals only, but purpose intact, an emphasis could be placed on part 3 (see Table 1) of the intervention. For example, someone’s purpose in life may be to become an Olympic champion in athletics. During the crisis, (s)he might not be able to pursue the intermediary goal to train three times a week at a running track. Though the purpose remains intact, the athlete should formulate new intermediate goals, for example, through an adapted scheme that focuses on an alternative and achievable training routine, which still allows the pursuit of the original purpose in a different way. For individuals with a compromised purpose in life, both part 2 and 3 would be important. For example, someone’s purpose in life may have been to build up a business and (s)he has just opened three restaurants. However, due to the pandemic and the restrictive measures, nobody can visit the restaurants, and therefore, the person loses the company. This person would need to think about a new purpose in life during and after the crisis, for it might take a while before the economy is fully restored, and opening new restaurants may be unrealistic in the near future. This person may have been very passionate about the hospitality business, and since purpose and significance in life are often intertwined, the sense of significance in life may also be compromised for this person. In such a case, it would be beneficial to take the full intervention, to discover new values and passions that lie within, and be able to find a new pathway to significance in life. This allows the person to discover other values and passions that exist besides the one that the person was focused on, and may help to find other directions in life that are also found worthy of pursuing.

**Table 1:** Elements and description of a life-crafting intervention.

Part	Elements	Tasks involved
1. Discovering values and passion	Values and passion	Writing about: (1) What they like to do, (2) what kind of relationships they would like to have, both in their private life and their work life, (3) what kind of career they would like to have, and (4) lifestyle choices
	Current and desired competencies and habits	(1) Qualities they admire in others, (2) competencies they have or would like to acquire, and (3) their own habits they like or dislike
2. Reflecting on one’s ideal future	Present and future social life	(1) Relationships that energize and de-energize them, (2) kinds of friends and acquaintances they would like to have in the future, and (3) what their ideal family life and broader social life would look like
	Possible future career (path)	(1) What is important in a job, (2) what is it they like to do, (3), what kind of colleagues do they want, and (4) whom do they want to meet through their work?
	Ideal vs. less ideal future	Best possible self and future when there are no (self-imposed) constraints. Contrast this with future if no changes are made
3. Writing about specific goal attainment and “if-then” plans	Goal attainment and “if-then” plans	(1) Formulating, strategizing, and prioritizing goals, (2) identifying and describing ways to overcome obstacles, and (3) monitoring progress toward goals
4. Making public commitment to the goals set	Public commitment to goal	Photo with statement, which communicates their goals to the world; communicating goals to friends, coworkers

Adapted from Schippers and Ziegler (2019).

## Discussion

Social and behavioral science research offers valuable insights into how the general population can be aided to cope better with the psychological effects of the COVID-19 pandemic and its restrictive measures (e.g., Schippers, 2020; Van Bavel et al., 2020). In this perspective paper, we argued that a life crafting intervention can be beneficial to rebuild meaning in life after it has been shattered by grief-like emotions over the loss of normalcy during the COVID-19 pandemic. A customized intervention is proposed based on the degree to which the sense of meaning has been affected.

An obvious advantage of the life-crafting intervention is that it is easily scalable. The expressive writing exercises can be done online, individually. This might be especially important during the COVID-19 pandemic, where many people struggle with psychological issues (e.g., Holmes et al., 2020), whereas demands on mental health care have increased, and are expected to maintain on a high level for the coming time. Some psychologists have argued that psychological help for the general population during this crisis has been largely overlooked (Van Hoof, 2020). Schippers (2020) has reviewed the combination of effects and ripple effects that the crisis and the measures that have been taken has in terms of economic, social, mental, and physical health, and presents a model of the interrelated effects. She also points to the fact that interventions are needed in order to counteract some of these effects. Fegert et al. (2020) expected that many young people will experience psychological problems not only during but also in the aftermath of the pandemic, and predict that the return to normality may take a long time.

The degree to which people suffer from psychological problems during the pandemic differs per individual and also depends on pre-existing psychological problems and vulnerabilities (e.g., Fegert et al., 2020). Therefore, there is even more need for customized, scalable interventions (see also Schippers, 2020). The large majority of the general population would likely not need extensive psychological care but could still benefit from interventions to rebuild their sense of meaning in life. For the more severe cases, more extensive psychological care would be needed. In a recent paper, it has been proposed that life-crafting can also be delivered using artificial intelligence, through a chatbot (Dekker et al., 2020). By using a chatbot, the intervention can be tailored to the individual’s needs, and can also be extended with other online psychological interventions aimed at improving mental health, such as cognitive behavioral therapy. We expect that such online tailored interventions would be sufficient for the large majority of the general population and could also be of (temporary) help for individuals with more severe problems, awaiting further professional psychological care.

To conclude, we propose that a life crafting intervention can help individuals to rediscover meaning in life, defined as a sense of purpose and significance (Martela and Steger, 2016), after this has been shattered in a grief-like situation. We expect that a renewed sense of meaning can help people cope with this collective trauma and hopefully resolve their grief over the loss of normalcy.

## Author Contributions

EJ, NZ, and MS equally contributed to the conceptual conception of the manuscript. EJ and NZ have written the draft of the manuscript. MS provided important intellectual input at all stages and reviewed and revised the manuscript. All authors contributed to the article and approved the submitted version.

### Conflict of Interest

The authors declare that the research was conducted in the absence of any commercial or financial relationships that could be construed as a potential conflict of interest.
